# Surgical Diseases of the Kidney

**Published:** 1887-03

**Authors:** 


Surgical Diseases of the Kidney. *By* Henry Morris, M.A., M.B., F.R.C.S., *Surgeon to and Lecturer on Surgery at the Middlesex Hospital, London. 12mo. Pp. 555, with 6 chromo-lithographic plates and 40 engravings. Philadelphia:* Lea Brothers & Company, 1886. *Chicago:* A. C. McClurg & Company.

There is no date upon either the title page or preface to show when this book was written. It is evident from the body of the work, however, that it was printed in 1886.

The coloring of the chromo-lithographic plates is much too high to be true to the appearances upon the surgical table or in the post-mortem room, but in this respect they are less at fault than such plates usually are. They have been carefully prepared, and they add much to the value of the volume.

Regarding the merits of the book, aside from the illustrations, it may be said that the author shows that he is fully at home in his treatment of the subject. The references to the literature involved are very abundant. The most striking defect in the book is the failure to sufficiently insist upon the importance of antiseptic details in the operations described. To this objection it is not enough to reply that the book is not written for beginners, for any book which does not insist upon the importance of careful and complete antiseptic measures in surgical operations will be condemned by many for the omission. E. W.

